# Higher performance for women than men in MRI-based Alzheimer’s disease detection

**DOI:** 10.1186/s13195-023-01225-6

**Published:** 2023-04-20

**Authors:** Malte Klingenberg, Didem Stark, Fabian Eitel, Céline Budding, Mohamad Habes, Kerstin Ritter, for the Alzheimer’s Disease Neuroimaging Initiative

**Affiliations:** 1 Charité - Universitätsmedizin Berlin (corporate member of Freie Universität Berlin, Humboldt-Universität zu Berlin, and Berlin Institute of Health), Department of Psychiatry and Neurosciences, Berlin, Germany; 2 Bernstein Center for Computational Neuroscience, Berlin, Germany; 3 Eindhoven University of Technology, Eindhoven, Netherlands; 4 Neuroimage Analytics Laboratory and Biggs Institute Neuroimaging Core, Glenn Biggs Institute for Neurodegenerative Disorders, University of Texas Health Science Center at San Antonio, San Antonio, TX USA

**Keywords:** Alzheimer’s disease, Deep learning, MRI, Sex, Bias

## Abstract

**Introduction:**

Although machine learning classifiers have been frequently used to detect Alzheimer’s disease (AD) based on structural brain MRI data, potential bias with respect to sex and age has not yet been addressed. Here, we examine a state-of-the-art AD classifier for potential sex and age bias even in the case of balanced training data.

**Methods:**

Based on an age- and sex-balanced cohort of 432 subjects (306 healthy controls, 126 subjects with AD) extracted from the ADNI data base, we trained a convolutional neural network to detect AD in MRI brain scans and performed ten different random training-validation-test splits to increase robustness of the results. Classifier decisions for single subjects were explained using layer-wise relevance propagation.

**Results:**

The classifier performed significantly better for women (balanced accuracy $87.58\pm 1.14\%$) than for men ($79.05\pm 1.27\%$). No significant differences were found in clinical AD scores, ruling out a disparity in disease severity as a cause for the performance difference. Analysis of the explanations revealed a larger variance in regional brain areas for male subjects compared to female subjects.

**Discussion:**

The identified sex differences cannot be attributed to an imbalanced training dataset and therefore point to the importance of examining and reporting classifier performance across population subgroups to increase transparency and algorithmic fairness. Collecting more data especially among underrepresented subgroups and balancing the dataset are important but do not always guarantee a fair outcome.

## Background

In recent years, a number of machine learning (ML) algorithms have been proposed to diagnose Alzheimer’s disease based on structural magnetic resonance imaging (MRI) [1, 2], and classification accuracies on par or even exceeding that of human experts have been reported [3]. One class of ML algorithms, namely convolutional neural networks (CNNs), have been shown to be very powerful for this task because they are capable of operating directly on raw or minimally processed MRI data and do not need a previous feature extraction [2, 4].

With the increasing use of ML methods in both medical and other decision-making systems, algorithmic bias, such as sex, gender, or racial bias, has come into the focus of current research. One possible cause for these biases is an underlying imbalance in the dataset used for training the ML algorithms. Medical research has, for a long time, been conducted primarily on male patients [5]. Additionally, even if female subjects are included, the results are often not analysed separately by sex or gender [6, 7]. This has, for example, led to underdiagnosis of heart attacks in women, as their symptoms can differ from those of male patients [8]. When unbalanced datasets are used to train ML algorithms, this can result in biased classifiers, with a consistent decrease in performance for population groups underrepresented in the training data [9, 10]. Several methods have been developed to understand and mitigate these biases. One simple and straightforward method is to ensure that the training datasets are balanced and representative across all relevant population subgroups. This can lead to a performance increase for underrepresented groups, while not necessarily negatively affecting performance for the overrepresented group [9, 11]. However, using a balanced dataset alone has been shown to not always be sufficient to prevent biased classification results as shown in a chest X-ray classification task recently [12]. Here, the differences in true-positive rate (TPR) across different subgroups are not correlated with the subgroups’ proportional disease membership and having the same portion of images within a label might not be enough to mitigate the resulting diagnostic bias [12].

From a clinical perspective, sex has an important impact on the presentation of AD. Women have a higher lifetime risk of developing AD, and also show faster ageing-related cognitive deterioration than men [13]. The atrophy rate both of the hippocampus and the overall brain matter is also higher for women than for men [14, 15]. Additionally, for women, pathological changes in the brain are more likely to result in clinical AD, with men being more resilient to the pathophysiological processes of AD [16, 17]. However, sex differences in the performance of ML-based classification of AD have so far not systematically been investigated.

Explainable artificial intelligence (AI) has become an important topic in recent years as more ML models are being implemented for medical applications [18]. For image data in combination with CNNs, the most promising approach refers to so-called heatmap or attribution methods that exploit the gradient or the architecture of the model to compute pixel- or voxel-wise explanations [19]. Notably, for each input image, a heatmap is generated that indicates the importance or relevance of each pixel or voxel for the final classification decision based on the respective model. In the medical context, it means that these methods provide a visual representation of the area that the model utilises for each individual patient, but do not provide any information on what is used within this area [4, 20].

In this study, we examine a state-of-the-art CNN classifier for MRI-based AD detection with respect to sex differences. To reduce the effects of possible biases in the training dataset, we balanced the training set to contain an equal number of women and men and used undersampling to equalise the female and male age distributions. We hypothesise that—in the case of balanced training data—there is no sex difference in detecting AD. To explain the classifier decisions, we use the Layer-Wise Relevance Propagation algorithm (LRP) [21], which produces an individual heatmap for each input image, showing the relevance of each voxel for the final classifier decision, and has been shown to give reasonable explanations in the context of AD [4, 22]. In particular, a significant correlation between local LRP relevance and atrophy in the hippocampus has been reported [4].

## Methods

### Data set

Data used in the preparation of this article were obtained from the Alzheimer’s Disease Neuroimaging Initiative (ADNI) database (adni.loni.usc.edu). The ADNI was launched in 2003 as a public-private partnership, led by Principal Investigator Michael W. Weiner, MD. The primary goal of ADNI has been to test whether serial magnetic resonance imaging (MRI), positron emission tomography (PET), other biological markers, and clinical and neuropsychological assessment can be combined to measure the progression of mild cognitive impairment (MCI) and early Alzheimer’s disease (AD).

#### Inclusion criteria

For this analysis, we included subjects from all ADNI study phases which were, at the time of their baseline visit, either healthy controls (HC) or diagnosed with AD using the official diagnoses provided by ADNI^1^. Subjects which were labelled as HC at their first visit, but at a later visit were diagnosed with either MCI or AD (or vice versa) were excluded from the analysis. This resulted in a population size of 573 subjects (406 HC, 167 AD). For each subject, up to three MRI scans from different time points were included in order to increase the sample size. To avoid data leakage between multiple scans originating from the same subject, we performed the splitting for the CNN training on the subject level and not on the image level (see below).

In the remaining population, younger female subjects are overrepresented [23]. To ensure that there is no significant difference in the age distributions of female and male subjects, we used undersampling based on subject age, diagnosis, and sex. To this end, we divided the population into bins containing subjects of a specific age range (in 5-year increments) and diagnosis (HC or AD). From each bin, we then randomly dropped subjects until the number of female and male subjects in the bin was equal. The resulting population contained 432 subjects (306 HC, 126 AD) with no significant differences in their age distributions (two-sample t-test: $p>0.75$). Table 1 gives full information about the size, age distribution, and clinical measures of the resulting dataset. The precise values vary slightly depending on the specific subjects removed during the undersampling step.

**Table 1:** Population characteristics. This table gives information about the demographics and clinical measures of one of the ten datasets created from the base study population. As different subjects are selected for each split, the values for other splits can vary slightly. All values are given as mean ± SD

	Female		Male	
	HC	AD	HC	AD
#subjects	153	63	153	63
#images	418	175	479	182
Age (years)	$73.8\pm 6.3$	$75.7\pm 7.3$	$73.8\pm 6.4$	$75.4\pm 6.9$
CDR-SB	$0.01\pm 0.07$	$4.63\pm 1.93$	$0.03\pm 0.15$	$4.68\pm 1.57$
ADAS13	$9.14\pm 4.49$	$30.58\pm 7.65$	$10.15\pm 4.53$	$31.56\pm 7.59$
MMSE	$29.17\pm 1.05$	$22.88\pm 2.54$	$28.99\pm 1.23$	$22.77\pm 2.04$

#### Splitting

We then used a stratified split based on subject age range, sex, and diagnosis to divide the population into a training set (216 HC, 90 AD) and validation and test sets (45 HC, 18 AD each). Splitting on the subject level ensures independence between training and test data, as splitting on the image level may lead to data leakage and unreliable results [2].

For datasets of this size, the results can vary significantly depending on the specific dataset split [23]. We therefore repeated the undersampling and splitting process ten times with different random seeds and trained and evaluated the classifier on all dataset splits. Depending on the number of scans taken of the subjects remaining after the undersampling, the training set size ranged from 758 to 834 images. For the test sets, we used only the baseline scan of each subject, to prevent the presence of multiple scans of the same subject distorting the results.

### Image preprocessing

For our analysis, we downloaded T1-weighted structural MRI scans of all selected subjects. The scans were acquired at multiple imaging sites at a magnetic field strength of 3 T (for scanner and sequence parameters, we refer the reader to the ADNI imaging protocols^2^). The images had already been preprocessed with gradient non-linearity correction (gradwarping) and intensity inhomogeneity correction and were scaled for gradient drift using the phantom data. We did not further harmonise the data.

We recently showed that, for a CNN trained on a relatively small dataset of MRI brain scans, using a non-linear registration method gives the best results compared to unregistered or linearly registered images [23]. Accordingly, we used the non-linear SyN algorithm [24] as implemented by Advanced Normalization Tools (ANTs)^3^ to register all scans to the 1mm T1 version of the MNI-ICBM152 reference brain. We chose SyN over other non-linear registration algorithms because of its consistently good performance reported by Klein et al. [25]. After registration, we used the FSL Brain Extraction Tool (fsl-bet) [26, 27] to remove the skull and soft tissue from the images.

### Network architecture and training

For this analysis, we used the convolutional neural network architecture proposed by Böhle et al. [4]. This is a standard CNN with four convolutional layers, each comprising 8, 16, 32, and 64 filters respectively, with a filter size of 3×3×3. Each of these layers is followed by batch normalisation and max pooling with window size 2, 3, 2, and 3. The convolutional layers are followed by two fully connected layers of sizes 128 and 2, with dropout ($p=0.4$) being applied before both of these layers. The 2-unit layer uses a softmax function and provides the model output, with the two units giving the class scores for HC and AD.

The network was trained using the Adam optimiser [28] and cross-entropy loss with a learning rate and weight decay of $10^{-4}$. We used a batch size of 16 images, which was limited by the available GPU memory. During training, the data was augmented by flipping the images across the sagittal plane and translating along the sagittal axis by up to two voxels in either direction, with both methods being standard data augmentation methods for deep learning methods performing medical image analysis [29, 30].

Training was stopped once the balanced accuracy achieved by the model on the validation set did not improve over eight epochs, after which the model state with the highest validation accuracy was evaluated on the test set. To achieve robust results and reduce the impact of lucky or unlucky data splits, we repeated the training process five times for each of the ten dataset splits, giving a total of 50 different models.

### Model evaluation and comparison to clinical measures

To evaluate whether there are statistically significant differences in the classifier performance for women and men, we used independent samples *t*-tests on the balanced accuracy, sensitivity, and specificity values. We also calculated the receiver operating characteristic (ROC) curves, which show the relationship between false-positive rate and true-positive rate, for women and men separately. This will allow to determine whether choosing different classification thresholds for women and men would help to achieve equal performance on the two subgroups.

We also examined the distributions of three different clinical measures of disease severity among men and women: the Clinical Dementia Rating (CDR) sum of boxes score [31], the Alzheimer’s Disease Assessment Scale (ADAS13) [32], and the Mini-Mental State Examination (MMSE) [33]. These measures have been shown to be correlated with both the brain atrophy rate and the ventricular enlargement rate [34] and can therefore provide insight into the degree of AD evidence present in the brain scans of our population. If there were a significant difference in the distributions of these measures between women and men, this could also explain a difference in classifier performance, as the differing disease severity could manifest as different degrees of structural AD evidence. To achieve robust results for this analysis, we used data from women and men, but only those subjects which appeared in at least two of the ten dataset splits.

### Layer-wise relevance propagation

For explaining the classifier’s decision, we used the layer-wise relevance propagation (LRP) algorithm by Bach et al. [21, 35], which produces heatmaps showing the relevance of each individual input voxel for the final classification.

To achieve this, LRP considers how the activation of each node in the model contributes to the final output class score layer by layer. The initial relevance value for a specific class is simply the activation of the corresponding output node. This relevance is then distributed to all nodes in the preceding layer which contributed to the activation of the output node. The distribution follows the conservation rule

1 $$\begin{aligned} \sum \limits _i R^{(l, l+1)}_{i\leftarrow j} = R^{(l+1)}_j \end{aligned}$$

where $R^{(l+1)}_j$ is the relevance of node *j* in layer $l+1$, and $R^{(l, l+1)}_{i\leftarrow j}$ is the share of relevance that node *i* in layer *l* receives from node *j*. The total relevance of a node in a specific layer is then the sum of the relevance it acquires from all nodes in the following layer. This ensures that the total amount of relevance in the input layer is precisely the output class score.

There are several different ways in which the relevance can be distributed through the model. For our analysis, we chose the $\beta$-rule [36], given by

2 $$\begin{aligned} R^{(l,l+1)}_{i\leftarrow j} = \left( (1+\beta )\frac{z_{ij}^+}{z_j^+} - \beta \frac{z_{ij}^-}{z_j^-} \right) R^{(l+1)}_j \end{aligned}$$

which allows for separate treatment of the positive and inhibitory contributions $z_{ij}^{+/-}$ by changing the parameter $\beta$. A value of $\beta = 0$ produces heatmaps showing only positive contributions (meaning evidence for the presence of AD), whereas choosing $\beta > 0$ includes inhibitory effects produced by evidence against AD. For a full description of the LRP algorithm and the $\beta$-rule, we refer the reader to Bach et al. [21] and Binder et al. [36].

When using LRP to visualise the decisions of an AD-detecting CNN, the resulting heatmaps are relatively robust to the chosen $\beta$-value, with the only change being increasing sparseness for higher values [4]. Additionally, ignoring negative contributions might give more informative results, as AD can, especially in its early stages, affect the brain in a highly localised manner. Surrounding healthy tissue could therefore mask the positive contributions of small areas of evidence for AD. We have accordingly limited our analysis to using a value of $\beta = 0$, as this leads to heatmaps showing all positive contributions, regardless of possible surrounding negative evidence, giving the final distribution rule

3 $$\begin{aligned} R^{(l,l+1)}_{i\leftarrow j} = \frac{z_{ij}^+}{z_j^+} R^{(l+1)}_j \end{aligned}$$

### Region-wise relevance analysis

To enable a quantitative analysis of the resulting heatmaps, we used the Neuromorphometrics Scalable Brain Atlas [37] to determine the amount of relevance present in the different brain regions. Simply summing the relevances of all voxels of a brain region naturally gives a measure that is strongly correlated to the region size [4]. We therefore normalised the relevance sum of each brain region by its number of voxels to determine its relevance density. This is a more informative measure, as a low amount of relevance spread out over a large area might be simply due to statistical noise, while a strongly localised cluster of voxels with high relevance could indicate the presence of structural changes as evidence for AD.

For the analysis, we selected a subset of brain regions which have been shown to have high susceptibility to structural changes due to AD. Among others, we included areas of the limbic system (such as the hippocampus, entorhinal area, and amygdala), the ventricles, and the cingulate gyrus. For comparison, we also included the motor cortex, which is among the last areas to be affected by AD [38].

## Results

### Classifier performance

**Fig. 1:**
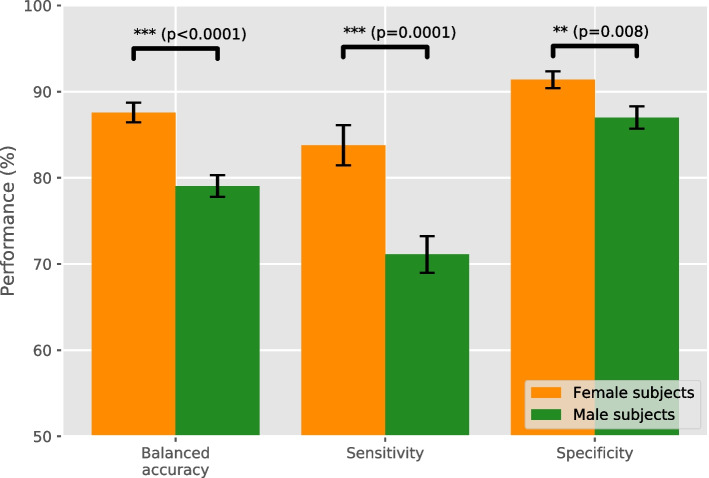
Classifier performance. The balanced accuracy, sensitivity, and specificity of the classifier for women and men averaged over all runs for all splits. The error bars show the standard error of the mean

The balanced accuracy, sensitivity, and specificity achieved by the classifier are shown in Fig. 1 for women and men separately. The given results are averaged over all 50 runs (five runs for each of the ten different dataset splits), with the error bars showing the standard error of the mean. A difference in performance for women and men is clearly visible, with a balanced accuracy of $87.58\pm 1.14\%$ for women and $79.05\pm 1.27\%$ for men. While the performance for women is better overall, the results seem to also be more robust, as determined by the lower standard error. A similar pattern holds for the sensitivity ($83.77\pm 2.34\%$ for women, $71.10\pm 2.13\%$ for men) and specificity ($91.38\pm 0.97\%$ for women, $86.99\pm 1.29\%$ for men). All differences are statistically significant, with *t*-test *p*-values of $p=2.4\cdot 10^{-6}$ for the balanced accuracy, $p=1.2\cdot 10^{-4}$ for the sensitivity, and $p=7.8\cdot 10^{-3}$ for the specificity.

**Fig. 2:**
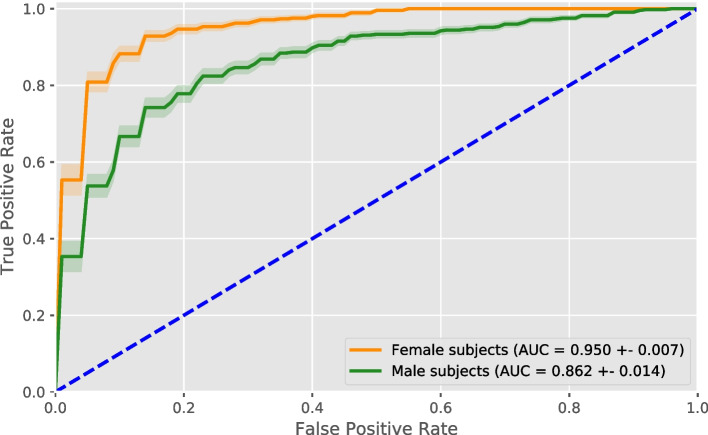
Receiver operating characteristic curve. The average ROC curve of the classifier when separately evaluated on women and men. The ROC curve was averaged over all runs for all splits, with the shaded area showing the standard error of the mean. The area under curve (AUC) is given in the legend

Figure 2 shows the ROC curves separately for women and men, averaged over all trained models. Again, a clear difference is visible, with an area under curve of $0.950\pm 0.007$ for women and $0.862\pm 0.014$ for men.

A post hoc analysis revealed that when we train a model on a sex-balanced dataset of half the size to generate a comparison baseline for sex-specific models, the classifier suffered a large drop in accuracy. Thus, due to the limited sample size, a subgroup analysis is not feasible here.

### Clinical measures

**Fig. 3:**
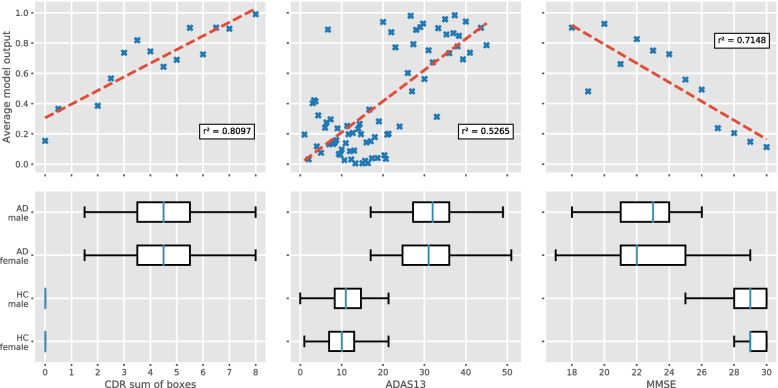
Clinical measures. Shown in the top row of plots is the relationship between the average model output and each of the three clinical measures (CDR sum of boxes, ADAS-Cog-13 and MMSE). While calculating the average output, only subjects appearing in at least two splits were taken into account. Overlaid in red is a linear regression, with the correlation coefficient also given. The plots on the bottom row show the distribution of the three clinical measures in the dataset. Note that, because this includes all subjects, the boxplot whiskers can extend past the values visible in the top plots

The distributions of the clinical AD measures in our dataset are presented in Fig. 3. The top row of plots show the relationship between the three clinical measures and the raw output of the classifier, i.e. after applying the softmax function. A clear correlation can be seen for all three measures, with scores indicating more severe disease leading to a higher model output.

The bottom row of plots gives the distribution of the clinical measures among women and men after balancing the dataset for age and sex. There are no significant differences between the CDR sum of boxes and MMSE scores of women and men for any of the ten dataset splits. The same is true for the ADAS13 scores of healthy subjects. For AD subjects, we found a significant difference in six out of the ten splits, but these differences were weak, with a *p*-score of $0.05>p>0.01$ in all of those cases but one ($p=0.006$).

### Visualisation

**Fig. 4:**
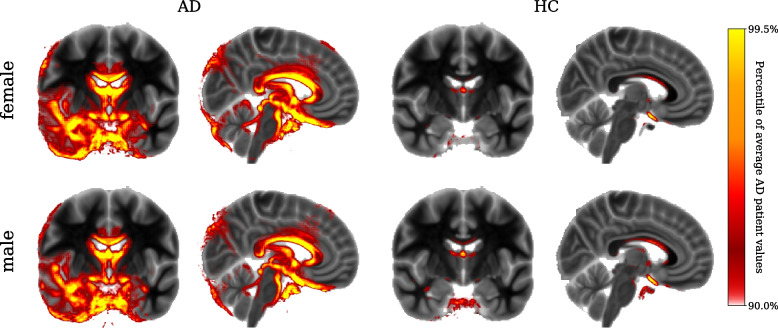
Average relevance heatmaps. The average relevance heatmaps across all subjects and all classifiers are shown separately for women (top row) and men (bottom row), as well as Alzheimer’s patients (left column) and healthy subjects (right column). The colour bar was chosen according to the relevance values of the average AD subject, with only the top 10% of values being shown to highlight the most relevant areas. For reference, the heatmaps are shown over the MNI-ICBM152 reference brain we used for registering the input images

In Fig. 4, we show the average heatmaps of all classifier decisions, separately for healthy and affected women and men. The heatmaps are overlaid over the MNI-ICBM152 template, only showing the top 10% of relevance values compared to the average AD heatmap. A coronal slice at $y=120$ shows areas of the frontal and temporal lobes, including the hippocampus and parahippocampal gyrus, and a sagittal slice at $x=85$ gives a view of, among others, the lateral ventricles, brain stem, and cerebellum.

For AD subjects, large amounts of relevance can be seen in and around the hippocampus and other areas of the temporal lobe. Significant relevance is also present around the lateral ventricle. There is only little relevance present in healthy subjects. No clear difference is visible between the average heatmaps for women and men.

**Fig. 5:**
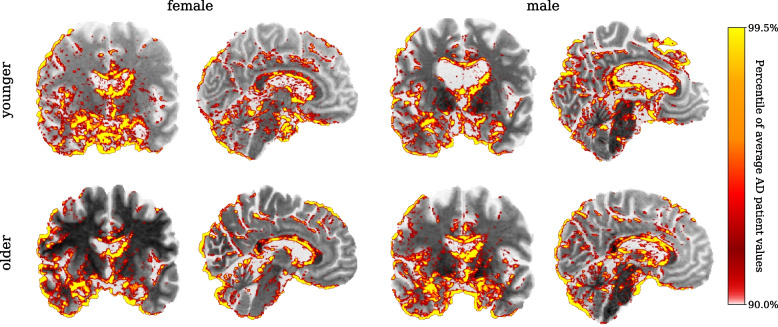
Individual relevance heatmaps. The relevance heatmaps for four individual AD patients are shown, each overlaid over the corresponding brain scan. All scans were classified by the same model, to enable a comparison of inter-subject differences. We selected two female (68 and 88 years) and two male subjects (67 and 87 years), which were correctly diagnosed by the classifier with high confidence (AD class score > 0.97). The colour bar was chosen as in Fig. 4, based on the relevance values of the average AD subject heatmap

To give a sense for the inter-patient variability of the heatmaps, we show the results for four individual subjects in Fig. 5. We selected four AD subjects, two women (68 and 88 years) and two men (67 and 87 years), and a single model which correctly classified all four subjects with high confidence (AD class score $>0.97$). It can be seen that the heatmaps are highly individual to each subject, although the general pattern of strong relevance in the temporal lobe and around the lateral ventricles still holds. The classifier also places significant amounts of relevance on individual cortices, which is well visible in all subjects but especially pronounced in the younger male brain, which shows severe atrophy. The younger male brain also shows a strong enlargement of the lateral ventricles. The classifier correctly identifies this, as can be seen by the relevance accurately placed on the border of the enlarged ventricle.

### Relevance analysis

**Fig. 6:**
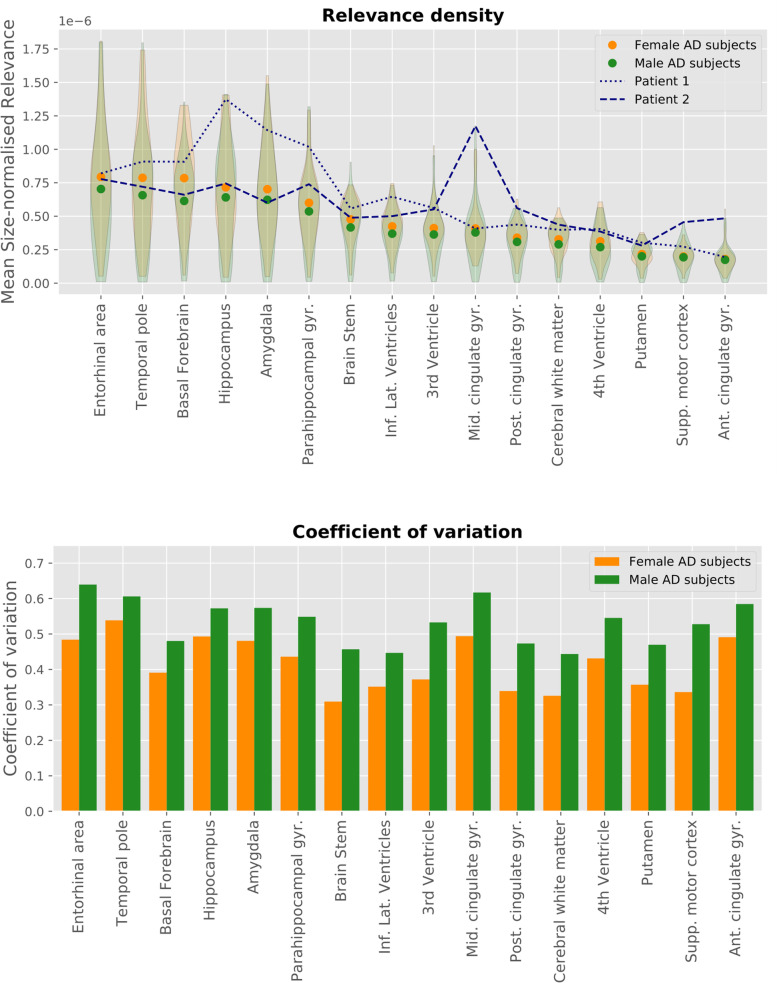
Relevance by area for AD subjects. The top plot shows the size-normalised relevance for selected brain areas for female and male AD subjects. The mean values are displayed as dots, with the shaded areas showing the relevance density distribution across all AD subjects. The dotted and dashed lines show the values for two individual subjects, namely the young female (Patient 1) and young male (Patient 2) subjects for which the heatmaps are shown in Fig. 5. The bottom plot gives the coefficient of variation, i.e. the standard deviation divided by the mean of the relevance density for the same brain areas

Figure 6 shows the relevance attributed by the LRP algorithm to several brain areas for female and male AD patients.

The highest relevance densities are found in areas that are part of the limbic system, such as the entorhinal area, the hippocampus, and the amygdala. The motor cortex, which is only affected in later stages of AD, has one of the lowest relevance densities among the examined regions.

The results for female and male patients are similar, with a close match in the order of area relevance density. However, the relevance density for women is consistently higher than for men.

We also show the results for two individual subjects, specifically the young female and male subjects from Fig. 5. This illustrates that the distribution of relevance over different brain areas can vary significantly between subjects. For the female subject, the relevance density is at the top of its range for hippocampus, parahippocampal gyrus, and amygdala, matching the visual impression of the heatmap.

The male patient has values around or slightly above average for most areas, except for the cingulate gyrus, which is among the highest values we found.

The bottom plot shows the coefficient of variation of the relevance density, i.e. its standard deviation divided by its mean. This measures the inter-patient variability of the relevance of the different brain areas. The coefficient is consistently larger for men by 10–30%; in other words, the distribution of relevance is more uniform for women than for men.

## Discussion

Despite balancing our dataset for subject sex and age, we found a statistically significant difference in classifier performance for women and men, with women having a higher balanced accuracy, sensitivity, and specificity. The classifier also achieves a higher area under the ROC curve for women, showing that the difference is not just due to a suboptimal threshold for men. There is no threshold value that would lead to equal performance for women and men, as their ROC curves do not intersect. Additionally, choosing individual thresholds for each population subgroup may not be a feasible solution in other, more complicated datasets with multiple intersectional subgroups and small subpopulations [10].

While an imbalance in the training data has been identified to be a possible cause of sex bias [9], other research has shown that the inverse is not true, with no significant correlation between classifier performance disparity and data imbalance ratio [12]. Our findings mirror this, as even a perfectly balanced dataset containing the same number of women and men does not lead to equal performance.

We have also examined a possible imbalance between women and men of disease severity as measured by several cognitive scoring systems. However, after equalising the age distributions, we did not find significant differences in disease severity for CDR and MMSE. The observed differences in ADAS13 scores for some splits indicated more severe cases in men, which would seem to indicate an easier diagnosis rather than the observed worse performance. While we have therefore excluded several possible sources of bias, we cannot rule out the presence of other confounding variables. Future research should investigate this further, looking for example at biomarkers of disease progression, such as the levels of amyloid beta and tau proteins. For example, Gamberger et al. have shown that when clustering the ADNI population based on biological and clinical descriptors, female AD patients form one coherent group, but male AD patients can be divided into two distinct clusters [39].

The observed performance difference may also be rooted in the different ways in which AD affects women and men. Female AD patients show both faster rates of cognitive decline and larger atrophy rates in limbic system areas specifically as well as overall brain matter when compared to male AD patients of the same age [14, 15, 40]. This implies that a perfect age-matching between women and men in the training data might actually have a detrimental effect in terms of classifier bias, as consistently more severe cases of AD in women would make them easier to diagnose. However, this hypothesis is contradicted by our analysis of the clinical measures of our study population, which showed no significant difference in disease severity between women and men.

On the other hand, men have been shown to have a higher resilience to the pathophysiological processes of AD compared to women [16, 17]. This means, that in cognitively intact older men, the presence of biomarkers for AD such as amyloid load has a lower influence on the clinical presentation than for women. As a consequence, the healthy male subjects in our study population might have more structural AD evidence than healthy female subjects, making the distinction between male healthy and AD subjects harder for the classifier. Because the increased amount of AD biomarkers does not lead to more severe cognitive symptoms in this case, this effect would not be visible in our analysis of clinical measures. Additionally, previous research has shown that men are at higher risk for cerebrovascular disease [41]. This could lead to higher prevalence in vascular cognitive impairment in men [42]. Even though the ADNI study inclusion criterion was AD-related dementia, dementia of vascular origin could be more present in the male cohort and might explain greater ventricle enlargement in men compared to women in our findings.

Regarding the heatmap results, our findings are in line with previous studies showing that LRP heatmaps are noisy and asymmetric [4, 22]. Moreover, a recent study showed that CNNs are susceptible to spatial bias, mainly due to architectural choices such as padding, resulting in activation blind spots in feature maps [43]. The stochastic nature of model training is expected to cause some randomness in the results; hence, the lack of symmetry is not surprising.

The heatmaps show that the classifier focuses on areas where structural changes due to AD are expected. Significant relevance being seen in the lateral ventricles in AD patients is reasonable as ventricular enlargement is a reliable measure of AD progression [44]. Additionally, the individual heatmaps agree with research showing that measures such as sulcal width and cortical thickness around sulci are good neuroanatomical markers of AD [45, 46]. Changes in periventricular white matter were shown to correlate with elevated cerebral amyloid [47] and cognitive decline [48]. Our findings that the relevance heatmaps focus on periventricular white matter could be capturing those clinical markers of AD. Our results showing relevance in the brainstem regions are in line with previous research showing brainstem atrophy in the early stages of AD [49, 50]. Overall, a visual inspection of the relevance heatmaps reveals no clear reason for the performance difference between women and men.The variability across subjects of different ages and between specific classifier models seems to eclipse any possible systematic difference between female and male heatmaps.

In the quantitative relevance analysis, the overall results are as expected from clinical research and match the visual impression of the presented heatmaps. The consistently higher relevance density for women compared to men indicates that the network was generally more confident when classifying women, as a higher model output results in a larger amount of relevance being distributed backwards through the network and thus a larger total relevance in the heatmap.

We found a larger variation in how the relevance is distributed among the brain areas for male AD subjects compared to female AD subjects. Additionally, the standard error of the performance metrics as given in Figs. 1 and 2 is slightly larger for men than for women, indicating a larger variation between different runs for male than for female subjects. This seems to reflect studies showing that there are general structural differences in the brains of women and men. These differences cover overall brain size, cortical thickness, and grey matter volume in specific brain areas, with men generally having a greater variance in these structural measures [51, 52]. However, other studies have questioned this, showing that, when corrected for brain size, sex accounts only for a small percentage of the structural variance, and that female and male brains are structurally very similar [53–55]. Men also have been shown to have more heterogeneous patterns of AD presentation compared to women. For instance, recent research has shown that the hippocampal sparing subtype of AD was more prevalent in men, which was associated with more white matter lesions [56].

We would like to point out the following limitations. First, we limited our analysis to only one specific network architecture, and thus, it is not clear to what extent these results will generalise to other classifiers, with either small changes such as an increase in the number of layers, or larger alterations to the entire network architecture. However, we used here a standard CNN that has been shown to be useful for AD classification before [4], and sex differences in classification accuracy have not yet been investigated.

Second, our dataset was quite small in terms of typical deep learning applications, with the training set size of around 800 images being limited by the available neuroimaging studies. Repeating our analyses on a larger dataset might alleviate the larger variance in male subjects. However, the ADNI study is currently the largest available dataset of brain MRI scans for AD, and the effect of larger male variance is already reduced due to our use of multiple dataset splits and several training runs per split. Given that deep learning analyses are very sensitive to the amount of training data, it was also not feasible to train sex-specific models (and models with different ratios of women/men) to additionally explore the influence of training set biases onto accuracy. Future studies might address this point using larger (not yet available) datasets.

Third, the preprocessing pipeline including skull stripping as well as the chosen brain atlas for quantitative analyses could be better adapted to the sample at hand by using age- and disease-specific templates [57, 58]. For instance, the high relevance density in the cingulate gyrus of the male patient from Fig. 5 is likely to be misattributed. As this patient has enlarged lateral ventricles, the boundaries of these ventricles do not match the brain atlas and instead extend into surrounding atlas areas. While the heatmap shows that the network correctly identifies the ventricle boundaries, the area-wise relevance analysis is based on the brain atlas and therefore can not take patient specifics into regard. However, disease-specific templates have the disadvantage to introduce prior information into the classification task, and thus might reduce the clinical significance of results. We therefore decided to keep the processing pipeline as general as possible.

And lastly, we did not employ an external validation dataset, instead generating both the training and validation datasets from the same study population (where we focused on clear AD and HC cases). This is common practice, as only few studies are testing the generalisability of classification models to external validation data, with results varying from only minor differences to comparatively large performance decreases [2, 59]. However, if the studies that collect the validation data adhere to the same inclusion criteria, the classifier performance would be similar to that on the initial dataset [2]. While independent and external validation of any classification and prediction models used in a healthcare setting is important before they are applied in clinical decision-making, the goal of our study was to point out performance differences between population subgroups. We believe that for this purpose, the cross-validation that we used by creating several different dataset splits was an appropriate choice. Yet, future studies should investigate how our findings apply across independent datasets, and with respect to changes in population (including for example MCI patients).

## Conclusion

In this study, we trained a CNN to detect AD on 3D MRI brain scans. Despite carefully balancing the training data for subject sex and age, we found that the classifier performs significantly better on women than on men. The difference was neither explainable by a suboptimal cutoff point, nor a difference in disease severity between women and men, as measured by cognitive assessments. We found some evidence indicating a higher variability among men, suggesting that controlling for subject sex and age might not be enough to ensure a truly balanced dataset. Even when accounting for other confounding variables, differing clinical manifestations of diseases for different population subgroups may make equal performance for all subgroups an unreachable goal. Collecting more data across subgroups and having a balanced dataset are important measures towards fairness of the algorithms; however, those measures are not always enough to provide a fair outcome. Therefore, when using ML methods in medical applications, care should be taken to evaluate and report bias in the resulting classifier. This would strengthen the transparency and fairness of the chosen methods and increase their chance of being adopted as diagnostic tools.

## Data Availability

The ADNI data base is public for researchers and can be downloaded upon request at https://adni.loni.usc.edu (see also the acknowledgements). The subject and image IDs for all used dataset splits and the code are available at https://github.com/malteklingenberg/AD-sex-bias.

## Footnotes

1 https://clinicaltrials.gov/ct2/show/NCT00106899

2 https://adni.loni.usc.edu/methods/documents/mri-protocols/

3 https://stnava.github.io/ANTs/
